# Genetic predictors of testosterone and their associations with cardiovascular disease and risk factors: A Mendelian randomization investigation^☆^

**DOI:** 10.1016/j.ijcard.2018.05.051

**Published:** 2018-09-15

**Authors:** C. Mary Schooling, Shan Luo, Shiu Lun Au Yeung, Deborah J. Thompson, Savita Karthikeyan, Thomas R. Bolton, Amy M. Mason, Erik Ingelsson, Stephen Burgess

**Affiliations:** a School of Public Health, The University of Hong Kong, Hong Kong Special Administrative Region; b School of Public Health and Health Policy, City University of New York, New York, NY, USA; c Centre for Cancer Genetic Epidemiology, Department of Public Health and Primary Care, University of Cambridge, Cambridge, Cambridgeshire, UK; d Cardiovascular Epidemiology Unit, Department of Public Health and Primary Care, University of Cambridge, Cambridge, Cambridgeshire, UK; e Division of Cardiovascular Medicine, Department of Medicine, Stanford University, Stanford, CA, USA; f MRC Biostatistics Unit, University of Cambridge, Cambridge, Cambridgeshire, UK; g Homerton College, University of Cambridge, Cambridge, Cambridgeshire, UK; h Stanford Cardiovascular Institute, Stanford University, Stanford, CA, USA

**Keywords:** BMI, body mass index, CAD, coronary artery disease, HDL, high-density lipoprotein, LDL, low-density lipoprotein, RCT, randomized controlled trial, SHBG, sex hormone-binding globulin, Testosterone, Sex hormones, Mendelian randomization, Causal inference, Genetic epidemiology, Disease aetiology

## Abstract

**Background:**

Testosterone supplementation has been linked to increased cardiovascular disease risk in some observational studies. The causal role of testosterone can be investigated using a Mendelian randomization approach.

**Methods and results:**

We assessed genetic associations of variants in two gene regions (*SHBG* and *JMJD1C*) with several cardiovascular risk factors (lipids, adiponectin, blood pressure, anthropometric traits) plus male pattern baldness, including control outcomes and potential mediators. We assessed genetic associations with coronary artery disease (CAD) risk in the CARDIoGRAMplusC4D consortium (171,191 individuals including 60,801 cases), and associations with CAD and ischaemic stroke risk in the UK Biobank (367,643 individuals including 25,352 CAD cases and 3650 ischaemic stroke cases).

Genetic predictors of increased serum testosterone were associated with lipids, blood pressure, and height. There was some evidence of an association with risk of CAD (*SHBG* gene region: odds ratio (OR) 0.95 per 1 unit increase in log-transformed testosterone [95% confidence interval: 0.81–1.12, p = 0.55]; *JMJD1C* gene region: OR 1.24 [1.01–1.51, p = 0.04]) and ischaemic stroke both overall (*SHBG*: OR 1.05 [0.64, 1.73, p = 0.83]; *JMJD1C*: OR 2.52 [1.33, 4.77, p = 0.005]) and in men. However, associations with some control outcomes were in the opposite direction to that expected.

**Conclusions:**

Sex hormone-related mechanisms appear to be relevant to cardiovascular risk factors and for stroke (particularly for men). However, the extent that these findings are specifically informative about endogenous testosterone or testosterone supplementation is unclear. These findings underline a fundamental limitation for the use of Mendelian randomization where biological knowledge about the function of genetic variants is uncertain.

## Highlights

• Genetic predictors of testosterone were associated with cardiovascular risk factors, such as lipids and blood pressure.
• However, associations were not consistent between the two gene regions considered in this analysis.
• Some genetic predictors of increased testosterone associated with higher coronary artery disease and ischaemic stroke risk.
• This suggests that some sex hormone-related mechanisms are implicated in cardiovascular disease.
• However, the extent to which these analyses are predictive of the effects of testosterone supplementation is unclear.

## 1 Introduction

Observationally low endogenous testosterone among men is associated with higher mortality [1] and increased risk of cardiovascular disease [2], although biases due to confounding by ill-health [3] cannot be ruled out. Testosterone replacement is increasingly prescribed for older men with age-related declines in testosterone, particularly in North America. Testosterone prescription has inconsistent associations with cardiovascular disease [4,5]; again, potential biases by indication, unmeasured confounding and time-related biases make interpretation of these observational studies uncertain. A study comparing the same individuals pre- and post-testosterone therapy to avoid these biases found a higher risk of non-fatal myocardial infarction with testosterone supplementation [6]. Evidence from randomized controlled trials (RCTs) about the effects of testosterone on cardiovascular disease and its risk factors is limited to small RCTs with limited follow-up time. Meta-analyses of RCTs of the effects of testosterone on cardiovascular disease in men have provided estimates in the direction of increased cardiovascular disease risk, but most have confidence intervals consistent with no effect [7]. The limited number of RCTs and incomplete reporting preclude any reliable estimates of the effect of testosterone on coronary artery disease or stroke. Meta-analysis of RCTs in men shows testosterone lowers high-density lipoprotein (HDL)-cholesterol [8] and adiponectin [9]. Meta-analyses of RCTs in women suggest testosterone lowers HDL-cholesterol and triglycerides while raising low-density lipoprotein (LDL)-cholesterol [10]. Warnings about cardiovascular risk of testosterone supplementation have been issued by Health Canada and the United States Food and Drug Administration, with Health Canada specifically mentioning that testosterone raises blood pressure and might cause blood clots. The Food and Drug Administration requested a trial to address the effects of testosterone supplementation on heart attack and stroke [7]. However, this will likely take many years before results are available.

In this situation, where observational evidence is potentially biased and evidence from RCTs is inadequate, naturally-occurring genetic variants can provide an alternative source of evidence for the presence of a causal effect [11,12]. Analyses of genetic variants specifically associated with a putative causal factor and their associations with a disease outcome are commonly referred to as Mendelian randomization [13]. Mendelian randomization studies have examined the association of genetic predictors of testosterone with cardiovascular disease and associated risk factors in men using genetic variants from the *FAM9B* (rs5934505), *SHBG* (rs12150660, rs1799941, rs6259), *CYP19A1* (rs10046, rs1008805) and *ESR2* (rs1256030) gene regions; none supported a causally protective effect of testosterone [[14], [15], [16], [17]] but instead indicated potential harms for blood pressure [14], lipids [15] and cardiac function [17]. To our knowledge, no previous Mendelian randomization investigating the effect on stroke has been undertaken. An intrinsic limitation for Mendelian randomization of sex hormones is that it is difficult to find genetic predictors of testosterone independent of sex hormone-binding globulin (SHBG). All the autosomal gene regions associated with testosterone concentrations at genome-wide significant levels [18,19] are also associated with SHBG [20], while gene regions on the X chromosome, such as the androgen receptor, have rarely been investigated.

To provide some insight into the role of testosterone in cardiovascular disease, we consider genetic predictors of serum testosterone established in genome-wide association studies and their associations with cardiovascular disease outcomes using the UK Biobank and published data from the largest available consortia for coronary artery disease (CAD), and for selected cardiovascular risk factors. Given meta-analysis of randomized controlled trials has shown that testosterone reduces HDL-cholesterol [8] and adiponectin [9], these were considered control outcomes which would be expected to have inverse associations with testosterone-increasing variants. Male pattern balding, associated with increased dihydrotestosterone, was also considered as a control outcome.

## 2 Methods

### 2.1 Genetic associations with testosterone

Genetic associations with log-transformed serum testosterone were obtained from 3225 men of European ancestry aged 50 to 75 years at increased risk of prostate cancer participating in the REDUCE study and consented to genetic studies on variants in two gene regions that were fine-mapped: 10q21 (*JMJD1C*, 661 variants) and 17p13 (*SHBG*, 325 variants) [19]. In the REDUCE study testosterone was measured at baseline using high turbulent flow liquid chromatography tandem mass spectrometry (Quest Diagnostics), with accuracy and precision that met the acceptance criteria [21]. Characteristics of men who did and did not consent to genetic studies were similar [19]. The mean testosterone level was 15.6 nmol/L, standard deviation 6.12 [19]. DNA samples were genotyped using the Illumina HumanOmniExpress Bead Chip. Quality control criteria for excluding SNPs included minor allele frequency < 0.01, call rate < 95% and p < 0.001 for the Hardy-Weinberg equilibrium test [19].

*JMJD1C* and *SHBG* are the only autosomal gene regions where variants have been demonstrated to be associated with testosterone at genome-wide significance. Lead variants in these gene regions explain about 1.1% (10q21) and 1.4% (17p13) of the variability in serum testosterone [19]. We did not include genetic variants from the Xp22 locus near *FAM9B*, also associated with testosterone at a genome-wide significance level [18], because genetic variants on sex chromosomes were not available in the consortium data analysed here. Genetic variants were filtered for validity in the UK Biobank. Variants associated with socio-economic position (education and Townsend index), or lifestyle factors (alcohol and smoking) at Bonferroni corrected significance, that deviated from Hardy Weinberg equilibrium at Bonferroni corrected significance, or were of poor imputation quality (info score < 0.6) were excluded from the analysis.

### 2.2 Genetic associations with cardiovascular disease risk factors

Genetic associations with HDL-cholesterol, LDL-cholesterol, and triglycerides were taken from the Global Lipids Genetics Consortium on up to 188,577 individuals of European ancestry [22]. Genetic associations with adiponectin were taken from the ADIPOGen Consortium on 29,347 individuals (discovery phase only) of European ancestry [23]. Genetic associations with blood pressure were obtained from the UK Biobank study on 367,643 individuals of European ancestry [24]. Overall and sex-specific genetic associations with body mass index (BMI) and height were taken from the Genetic Investigation of Anthropometric Traits (GIANT) consortium on up to 322,154 (BMI) [25] and 253,288 (height) [26,27] individuals of European ancestry. Genetic associations with male pattern balding were estimated in the UK Biobank study on 112,362 men of European descent. Balding pattern was self-recorded, and coded as no balding (full head of hair) versus partial balding or majority or complete balding.

### 2.3 Genetic associations with cardiovascular disease outcomes

Genetic associations with CAD risk were obtained from the Coronary Artery DIsease Genome wide Replication and Meta-analysis plus Coronary Artery Disease (CARDIoGRAMplusC4D) consortium. In CARDIoGRAMplusC4D, genetic associations were available for a maximum of 171,191 total participants mostly of European ancestry with some participants of South Asian ancestry including up to 60,801 incident CAD cases [28]. The overall analyses for CARDIoGRAMplusC4D used a 1000 Genomes reference panel [28]. Overall and sex-specific genetic associations with CAD and ischaemic stroke risk were obtained from the UK Biobank; genetic associations were available for a maximum of 367,643 unrelated participants of European ancestry including 25,352 CAD cases and 3650 stroke cases.

### 2.4 Statistical analysis

For each risk factor and outcome, we performed analyses using summarized data that are equivalent to constructing a weighted genetic risk score for the risk factor using the individual-level data, and then assessing the associations of this score with the outcome [29]. The summarized data analyses were undertaken by generalized weighted linear regression of the genetic associations with the outcome on the marginal (univariate) genetic associations with the risk factor, using inverse-variance weights from the genetic associations with the outcome, setting the intercept to zero [30], and using population-specific correlations between the variants obtained from LDlink using the 1000 Genomes Project (Phase 3). All analyses were performed separately for the two gene regions. The statistical model was:

$$\beta_{\mathrm{OUTCOME},j}=\theta \;\beta_{\mathrm{RISKFACTOR},j}+\varepsilon_{j},\mathrm{Var}\left(\varepsilon \right)=\Sigma ,\Sigma_{j_{1}j_{2}}=\mathrm{se}\left(\beta_{\mathrm{RISKFACTOR},j_{1}}\right)\mathrm{se}\left(\beta_{\mathrm{RISKF}A\mathrm{CTOR},j_{2}}\right)\rho_{j_{1}j_{2}}$$

where *β*_*OUTCOME*, *j*_ is the estimated association with the outcome for variant *j*, *β*_*RISKFACTOR*, *j*_ is the estimated association with the risk factor for variant *j*, *θ* is the Mendelian randomization estimate, Σ is the variance-covariance matrix of the error terms in the regression, and *ρ*_*j*_1_*j*_2__ is the correlation between variants *j*_*1*_ and *j*_*2*_. For variants in the SHBG gene region, principle components explaining 99% of the variance of the weighted correlation matrix were used to stabilize estimates as described previously [31].

Analyses were performed using the *MendelianRandomization* package in the R software platform (v3.4.3 “Kite-Eating Tree”).

Two-sided p-values are reported throughout, with correction for multiple testing using a p-value of 0.05/7 = 0.007, given the two disease outcomes (CAD, and stroke) and four classes of cardiovascular risk factors (lipids, adiponectin, blood pressure, anthropometric measures) plus male pattern balding. This level of correction provides a balance between rigorous results, and avoiding false negatives, particularly given the generally low power of Mendelian randomization investigations, and the lack of prior justification for favouring the null hypothesis (no causal effect). Power calculations were performed for a range of sample sizes using online tools [32].

### 2.5 Choice of genetic variants

We considered two separate approaches for selecting genetic variants in the analysis: a stepwise selection approach and a conditional analysis approach. We performed a stepwise selection by taking the genetic variant having the strongest association (smallest p-value) with testosterone from that gene region, then adding the next strongest variant not in strong linkage disequilibrium with the initial variant (r^2^ < 0.4), and so on to give a set of variants with low pairwise correlations. Different sets of variants were used for the analysis of each outcome, as the genetic variants available differed.

As an alternative approach, we also performed Mendelian randomization analyses using a genetic score comprising of variants associated with SHBG in a previous conditional analysis of 13,899 women and 14,938 men mostly of European ancestry [20]. After filtering for validity in the UK Biobank, no variants remained in the 10q21 gene region, and up to 8 variants in the 17p13 gene region. The precise choice of variants for each analysis is indicated in Supplementary Tables 1–3.

This investigation uses published or publicly-available data and the UK Biobank only. No original data were collected for this manuscript. Ethical approval for each of the studies included in the investigation can be found in the original publications (including informed consent from each subject). The study protocol conforms to the ethical guidelines of the 1975 Declaration of Helsinki.

## 3 Results

Genetic associations with testosterone and SHBG were concordant in direction for all reported variants, and also concordant in sex-specific analysis with SHBG for both men and women [20]. Genetic associations with dihydrotestosterone were also concordant in direction [19]. Hence, the Mendelian randomization estimates should not be thought of as exclusively relating either to SHBG or to testosterone, but reflect changes in both testosterone and SHBG. Power calculations for a range of effect sizes are in Supplementary Fig. 1; power to detect a risk ratio of 1.2 per 1-standard deviation change in the exposure was 93.4% for CAD and 47.9% for stroke.

### 3.1 Genetic associations with cardiovascular risk factors

Estimates from Mendelian randomization analyses for lipids, adiponectin, blood pressure, anthropometric traits, and male pattern balding are displayed in Table 1 for variants in the 10q21 (*JMJD1C*) gene region, and Table 2 for variants in the 17p13 (*SHBG*) gene region for the stepwise score and Supplementary Table 4 for the conditional score.

**Table 1:** Mendelian randomization estimates for the effect of testosterone on cardiovascular risk factors using variants in 10q21 (*JMJD1C*) gene region.

Outcome	Estimate (SE)	p-Value
HDL-c	0.085 (0.068)	p = 0.21
LDL-c	**0.293** (**0.073**)	**p** < **0.0001**
Triglycerides	−**0.229** (**0.066**)	**p** < **0.0001**
Adiponectin	0.103 (0.070)	p = 0.14
Systolic blood pressure	−**2.236 (0.578)**	**p** < **0.0001**
Diastolic blood pressure	−**1.644 (0.331)**	**p** < **0.0001**
BMI	−0.055 (0.056)	p = 0.33
BMI (men)	0.017 (0.074)	p = 0.82
BMI (women)	−0.120 (0.070)	p = 0.084
Height	0.059 (0.046)	p = 0.20
Height (men)	0.135 (0.097)	p = 0.16
Height (women)	−0.065 (0.089)	p = 0.47
Male pattern balding	−0.051 (0.125)	p = 0.69

Mendelian randomization estimates for high-density lipoprotein (HDL) cholesterol, low-density lipoprotein (LDL) cholesterol, triglycerides (all SD units), adiponectin (μg/ml, log-transformed), systolic and diastolic blood pressure (mmHg), body mass index (BMI, SD units) and height (SD units), and male pattern baldness (log odds ratio, positive values favour more balding). Estimates (standard errors) are changes in the outcome per unit increase in log-transformed testosterone (stepwise score). Genetic associations were measured in men and women unless otherwise indicated. Italics indicates associations at a nominal level of significance without correction for multiple testing (p < 0.05), bold indicates associations after correction for multiple testing (p < 0.05/7 = 0.007).

**Table 2:** Mendelian randomization estimates for the effect of testosterone on cardiovascular risk factors using variants in 17p13 (*SHBG*) gene region.

Outcome	Estimate (SE)	p-Value
HDL-c	**0.145** (**0.042**)	**p** = **0.001**
LDL-c	−**0.143** (**0.046**)	**p** = **0.002**
Triglycerides	−**0.155** (**0.041**)	**p** < **0.0001**
Adiponectin	**0.114** (**0.039**)	**p** = **0.003**
Systolic blood pressure	**1.350 (0.448)**	**p** < **0.0001**
Diastolic blood pressure	**1.154 (0.257)**	**p** < **0.0001**
BMI	0.057 (0.031)	p = 0.068
BMI (men)	*0.098* (*0.042*)	p = *0.02*
BMI (women)	0.026 (0.039)	p = 0.503
Height	**0.254** (**0.025**)	**p** < **0.0001**
Height (men)	**0.293** (**0.044**)	**p** < **0.0001**
Height (women)	0.024 (0.041)	p = 0.562
Male pattern balding	0.013 (0.082)	p = 0.89

Mendelian randomization estimates for high-density lipoprotein (HDL) cholesterol, low-density lipoprotein (LDL) cholesterol, triglycerides (all SD units), adiponectin (μg/ml, log-transformed), systolic and diastolic blood pressure (mmHg), body mass index (BMI, SD units) and height (SD units), and male pattern baldness (log odds ratio, positive values favour more balding). Estimates (standard errors) are changes in the outcome per unit increase in log-transformed testosterone (stepwise score). Genetic associations were measured in men and women unless otherwise indicated. Italics indicates associations at a nominal level of significance without correction for multiple testing (p < 0.05), bold indicates associations after correction for multiple testing (p < 0.05/7 = 0.007).

#### 3.1.1 Cardiovascular risk factors

Variants in the 10q21 gene region were negatively associated with triglycerides and blood pressure, but positively associated with LDL-cholesterol. Variants in the 17p13 gene region were positively associated with HDL-cholesterol and blood pressure, with negative associations with triglycerides and LDL-cholesterol for the stepwise genetic score. The association with adiponectin in the 10q21 gene region was null, but the association for the stepwise genetic score in the 17p13 gene region was positive.

#### 3.1.2 Anthropometric traits

Variants in the 10q21 gene region had little association with BMI or height. Variants in the 17p13 gene region had some association with BMI in men, and were positively associated with height in men.

#### 3.1.3 Male pattern balding

All associations with male pattern balding were null.

### 3.2 Genetic associations with cardiovascular disease outcomes

Estimates from Mendelian randomization analyses for the cardiovascular disease outcomes are displayed in Table 3 for the stepwise scores and Supplementary Table 4 for the conditional score. Variants in the 10q21 gene region were associated with increased risk of CAD in CARDIoGRAMplusC4D, but not in UK Biobank. Variants in the 17p13 gene region were not associated with risk of CAD.

**Table 3:** Mendelian randomization estimates for the effect of testosterone on cardiovascular disease outcomes using the stepwise score for variants in the 10q21 (*JMJD1C*) and 17p13 (*SHBG*) gene regions.

Gene region:	10q21 (*JMJD1C*)		17p13 (*SHBG*)	
CAD (overall, UKBB)	0.96 (0.75 to 1.23)	p = 0.75	0.98 (0.80 to 1.18)	p = 0.81
CAD (men, UKBB)	1.04 (0.72 to 1.50)	p = 0.83	0.98 (0.74 to 1.31)	p = 0.91
CAD (women, UKBB)	0.89 (0.64 to 1.25)	p = 0.51	0.97 (0.74 to 1.26)	p = 0.81
CAD (overall, C + C4D)	**1.46** (**1.14 to 1.87**)	**p** = **0.003**	0.95 (0.77 to 1.16)	p = 0.58
CAD (overall, UKBB and C + C4D)	*1.24* (*1.01 to 1.51*)	p = *0.038*	0.95 (0.81 to 1.12)	p = 0.55
Ischaemic stroke (overall, UKBB)	**2.52 (1.33 to 4.77)**	**p** = **0.005**	1.05 (0.64 to 1.73)	p = 0.83
Ischaemic stroke (men, UKBB)	**3.18** (**1.43 to 7.05**)	**p** = **0.004**	1.67 (0.90 to 3.11)	p = 0.10
Ischaemic stroke (women, UKBB)	1.63 (0.56 to 4.73)	p = 0.37	0.46 (0.20 to 1.05)	p = 0.066

Mendelian randomization estimates for coronary artery disease (CAD) and ischaemic stroke in UK Biobank (UKBB) and publicly available data, i.e., CARDIoGRAMplusC4D 1000 Genomes-based GWAS (C + C4D). Estimates (95% confidence intervals) are odds ratios per unit increase in log-transformed testosterone from overall and sex-specific analyses. Italics indicates associations at a nominal level of significance without correction for multiple testing (p < 0.05), bold indicates associations after correction for multiple testing (p < 0.05/7 = 0.007).

**Fig. 1:**
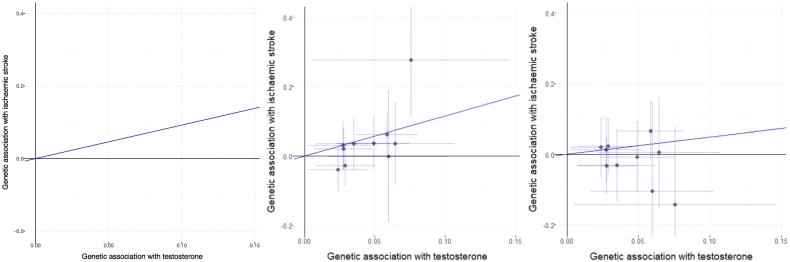
Genetic associations with ischaemic stroke against genetic associations with testosterone. Genetic variants are those in the 10q21 (*JMJD1C*) gene region included in the stepwise selection score. Genetic associations with testosterone (per allele increase in log-transformed testosterone) were estimated in men only and are taken from Jin et al. [19]; genetic associations with ischaemic stroke risk (per allele log odds ratio) are taken from the UK Biobank and were estimated in men and women (left), men only (middle), and women only (right).

Variants in the 10q21 gene region were associated with a higher risk of stroke in men. Overall associations with risk of stroke for variants in the 17p13 gene region were null, although there were opposite directions of association in the sex-specific analyses. Genetic associations with testosterone and with stroke, both overall and sex-stratified, are displayed in Fig. 1 for the variants included in the stepwise score for the 10q21 gene region.

## 4 Discussion

Genetic predictors of testosterone from the 10q21 (*JMJD1C*) and 17p13 (*SHBG*) gene regions were clearly negatively associated with triglycerides and for 17p13 were positively associated with HDL-cholesterol and adiponectin. The associations with LDL-cholesterol and blood pressure differed in direction between the two regions. Some evidence was observed of genetic predictors of testosterone in the 10q21 gene region being associated with higher CAD risk, and with a higher risk of ischaemic stroke in men. The issue is the extent to which these findings correspond with the effects of endogenous or exogenous testosterone.

Meta-analysis of RCTs suggests exogenous testosterone raises LDL-cholesterol, lowers HDL-cholesterol and lowers triglycerides in women [10], lowers HDL-cholesterol in men [8] and lowers adiponectin in men [9]. Meta-analysis of RCTs also suggests the anti-androgen finasteride, which targets the testosterone metabolite dihydrotestosterone, is an effective treatment for male pattern balding [33]. As such, the results of this genetic investigation have somewhat limited correspondence with the known effects of exogenous testosterone – in fact, using 17p13 (*SHBG*) variants association estimates of testosterone with LDL-cholesterol, HDL-cholesterol, triglycerides and adiponectin are in the opposite directions from the RCT findings [[8], [9], [10]]. Examples where the naive Mendelian randomization estimates using circulating levels of the biomarker are in the opposite direction from the biologically validated causal effect have previously been observed for interleukin-6 [34] and for extracellular superoxide dismutase [35].

One possible explanation is that our findings relate to the effects of lower endogenous testosterone action, because the associated higher SHBG reduces bio-available sex hormones. Alternatively, effects of endogenous and exogenous testosterone may differ, although that is perhaps implausible. Response to testosterone supplementation in hypogonadal men is also quite variable [36]. Testosterone supplementation in mature men may not have the same effects as the lifelong differences in testosterone concentrations considered in Mendelian randomization investigations; particularly given the role of testosterone in growth and development. Finally, circulating serum testosterone might not correspond to total testosterone [37], including locally synthesized testosterone, which may also play a role in cardiovascular disease and which observationally has associations with cardiovascular disease and its risk factors that are more consistent with findings from RCTs than those of serum testosterone [38]. However, there were differences in genetic associations between men and women for some outcomes, which would be consistent with a causal role of sex hormone-related mechanisms. Additionally, a dose—response relationship between genetic associations with testosterone and stroke risk for variants in the 10q21 gene region, which again is consistent with a causal explanation.

These results could also represent effects beyond those of increased endogenous testosterone and SHBG. Previous Mendelian randomization studies of SHBG have largely used genetic variants from the *SHBG* gene region, and so also suffer from the same lack of specificity. Null, but concordant associations with CAD were present in women; it is perhaps unlikely that such associations would be solely driven by testosterone, as testosterone levels in women are much lower than those in men. There was evidence for sex-specific differences in genetic associations with height, BMI and stroke. Associations with height for variants in the 17p13 region were stronger for men. The genetic associations with cardiovascular disease risk factors (in particular, for LDL-cholesterol and blood pressure) were heterogeneous between the two gene regions considered, meaning that it is impossible that both gene regions are good proxies for testosterone supplementation. The 10q21 gene region is also known to be associated with white blood cell count [39], vascular endothelial growth factor [40], platelet aggregation [41], QRS duration [42], and liver function [43]. It not clear whether these associations of the gene regions are pleiotropic in nature (that is, the gene region affects multiple independent pathways), or whether they represent mediation (these associations may reflect downstream consequences of testosterone variation rather than true pleiotropy) or effects of some upstream precursors of testosterone. Testosterone is known to affect platelet aggregation and endothelin-1, which have recently been implicated in CAD by Mendelian randomization studies [44,45].

This investigation has several strengths as well as weaknesses. First, regarding the choice of data, the largest available published sources of genetic associations with testosterone and SHBG were used, which were then filtered for validity using the UK Biobank. Equally, the largest available published sources of genetic associations with CAD risk were used. Generally, the investigations were well powered to detect moderate effect sizes. Sex-specific genetic association estimates were obtained from the UK Biobank, allowing differences in associations between men and women to be compared. The genetic associations with sex hormones were estimated in different participants from the genetic associations with outcomes. This reduces the potential effect of winner's curse to give false positive results. Genetic associations were estimated mostly in European descent participants, and so the results may not be applicable to those of other ethnicities. Notably variants in the *JMJD1C* associated with testosterone in older men at increased risk of prostate cancer did not replicate in younger, healthy Chinese men (mean age 37.4 years) [46]. Although stratification by ethnicity and adjustment for principal components were performed, some of the consortia also included individuals of other ethnicities. We were unable to obtain sex-specific genetic association estimates for all the outcomes. We were unable to use exactly the same definition CAD in UK Biobank and CARDIoGRAMplusC4D, which may explain the different estimates. In both gene regions, some heterogeneity in associations with the outcomes existed for different variants within the gene regions, meaning that somewhat different results could be obtained by different choices of genetic variants. However, Mendelian randomization estimates for CAD using variants in the 17q13 (*SHBG*) gene region were similar when genetic variants were chosen as predictors of testosterone or SHBG, suggesting that result is somewhat robust to choice of variants. This finding corroborates a previous Mendelian randomization investigation for myocardial infarction using a variant (rs1799941) in the *SHBG* gene region [16], in the CARDIoGRAMplusC4D consortium, this variant was negatively associated with myocardial infarction risk (p = 0.046). While the overall results for stroke were null, this may indicate a lack of power rather than a genuine null result; there was evidence for an effect on stroke risk in men. We were also unable to identify valid genetic predictors of SHBG in the 10q21 gene region. Finally, the weights in the gene scores for testosterone were derived in men only. Although a GWAS investigation for testosterone in women has been performed, no regions were associated at a genome-wide level of significance [47]. This means that the analyses may have limited relevance for women, although associations with testosterone were concordant with associations with SHBG for both men and women for all variants for which data were available [20].

Limited experimental evidence concerning the effects of testosterone makes it difficult to contextualize these findings. To date, the effects of testosterone on cardiovascular disease in men have rarely been investigated, perhaps because of the tenacity of the assumption that higher rates of cardiovascular disease in men than women are due to oestrogen in women. The published literature also suffers from citation bias in favour of testosterone [48]. Applying theories from evolutionary biology, growth and reproduction would be expected to trade-off against longevity [49]. Consistent with this theory, evidence of genetic selection in favour of both fertility and CAD has recently been reported [50]. A recent Mendelian randomization study also found some indications that genetically-predicted higher follicle stimulating hormone, usually associated with higher androgen levels, was positively associated with CAD risk, but genetically-predicted higher anti-Müllerian hormone and higher risk of testicular dysgenesis syndrome, usually associated with lower androgen levels, were negatively associated with CAD risk [51].

In conclusion, this investigation suggests that sex hormone-related mechanisms have a role for both cardiovascular risk factors (in particular, lipids, blood pressure, and anthropometric measurements), and for CAD and for stroke (particularly for men). We have employed state-of-the-art methods and the largest currently-available data resources to answer the question of causality as comprehensively as possible using human genetics. Further biological insight of the variants' function, or additional variants more specifically associated with testosterone would be required to assess the extent to which the effects of intervention on a specific sex hormone (such as testosterone supplementation or inhibition) can be predicted using autosomal genetic variation in serum testosterone, as well as to inform whether supplementation is likely to increase or disease cardiovascular disease risk. This tentative conclusion represents the current limit of what can be learned using a Mendelian randomization approach with these variants.

## Sources of funding

No specific funding was received for the writing of this manuscript. Stephen Burgess is supported by a Sir Henry Dale Fellowship jointly funded by the Wellcome Trust and the Royal Society (Grant Number 204623/Z/16/Z). This work was partly supported by a small project fund (#201409176231) from the University of Hong Kong.

## Disclosures

Erik Ingelsson is an advisor and consultant for Precision Wellness, Inc., and advisor for Cellink for work unrelated to the present study. There are no conflicts of interest relating to this manuscript.

## Appendix A Supplementary data

Supplementary Methods and Results. Image 1
