# Supplementary material for: Genetic predictors of testosterone and their associations with cardiovascular disease and risk factors: A Mendelian randomization investigation
Source: Int J Cardiol. 2018 Sep 15;267:171–6. doi: 10.1016/j.ijcard.2018.05.051 (PMC6024225; doi:10.1016/j.ijcard.2018.05.051)
Supplement: Supplementary file 1 — Supplementary Methods and Results. [file mmc1.docx]

**Supplementary material:**

S1 Supplementary Methods. Genetic association estimates in UK Biobank.

Supplementary Figure 1: Power calculations for binary and continuous outcomes

Supplementary Table 1: Genetic variants used in the Mendelian randomization analyses for the conditional analysis scores

Supplementary Table 2: Genetic variants in the 10q21 gene region used in the Mendelian randomization analyses for the stepwise selection scores

Supplementary Table 3: Genetic variants in the 17p13 gene region used in the Mendelian randomization analyses for the stepwise selection scores

Supplementary Table 4: Mendelian randomization estimates for the effect of sex hormone-binding globulin on cardiovascular risk factors, coronary artery disease risk and ischemic stroke risk using variants for the conditional (SHBG) score in 17p13 (*SHBG*) gene region

**S1 SUPPLEMENTARY METHODS. Genetic association estimates in UK Biobank.**

Genetic association estimates were all obtained from the literature, with the exception of associations with blood pressure, male-pattern baldness, CAD and ischemic stroke. These were estimated in the UK Biobank cohort, in 367,643 individuals (blood pressure, CAD and ischemic stroke) and 112,362 men (male-pattern baldness) who passed quality control checks and were of European ancestry (judged by self-report and by genomic principal components). Extensive quality control procedures were used to clean the dataset. Individuals were excluded from the analysis if they were sex mismatches, had excess heterozygosity (>3 standard deviations from the mean), or were related to another individual as a third-degree relative or closer (one of each pair of related individuals was removed at random). Associations estimates were estimated in the *snptest* software package (https://mathgen.stats.ox.ac.uk/genetics_software/snptest/snptest.html) by linear or logistic regression with adjustment for age, sex, and 10 genomic principal components.

**Supplementary Figure 1** Power curves for binary and continuous outcomes: (left) binary outcome for sample size of 170k with 60k cases [blue line, representing CAD as outcome in CARDIoGRAMplus C4D1000G], and sample size of 367.6k with 8.4k cases [red line, representing ischemic stroke as outcome in the UK Biobank]; (right) continuous outcome for sample size of 29k [blue line, representing adiponectin as outcome in ADIPOGen], and sample size of 346.7k [red line, representing blood pressure as outcome in UK Biobank].

All power calculations presented assume the genetic variants explain 1.1% of the variance in the risk factor. This is likely to be a conservative estimate for most analyses, as the lead variant alone explained 1.1% (10q21) and 1.4% (17p13) of the variability in serum testosterone in previous analyses. The left panel represents the power with a binary outcome for a risk ratio effect size of between 1.0 (null effect) and 1.5 per 1 standard deviation change in the risk factor. The blue line represents power in the CAD analyses using genetic associations from CARDIoGRAMplusC4D1000G, whereas the red line represents power in the stroke analyses using genetic associations from the UK Biobank. The right panel represents the power with a continuous outcome for an effect size of between 0 (null effect) and 0.03 standard deviation units in the outcome per 1 standard deviation change in the risk factor. The blue line represents power in the blood pressure analyses using genetic associations from UK Biobank, whereas the red line represents power in the adiponectin analyses using genetic associations from ADIPOGen. These are the smallest datasets that genetic associations with continuous outcomes are estimated in. Despite these conservative assumptions, power is reasonable (above 80%) in all analyses with a continuous outcome for an effect size of around 0.15, and with a binary outcome for an effect size of 1.15 for CAD and 1.3 for ischemic stroke.

Associations (standard errors) with sex hormone binding globulin (SHBG) and testosterone (T) are the changes in the log-transformed sex hormone per additional copy of the effect allele; associations are taken from Coviello *et al* (SHBG) and from Jin *et al* (T). A tick indicates that the variant was used in the analysis for that risk factor. EA, effect allele; OA, other allele; EAF, effect allele frequency.

^1^ rs12150660 is highly correlated (r^2^ = 0.92) with rs1799941, a variant that has been used in several previous Mendelian randomization studies for SHBG.
^2^ The sex-specific associations with SHBG of rs12150660 are 0.110 (0.006) in men and 0.087 (0.008) in women. Sex-specific associations were not available for other variants.

Supplementary Table 4: Mendelian randomization estimates for the effects of sex hormone-binding globulin on cardiovascular risk factors, and coronary artery disease and ischemic stroke risk using variants for the conditional score in 17p13 (*SHBG*) gene region

| Cardiovascular risk factors and control outcomes |  |  | Estimate (SE) p-value |
| --- | --- | --- | --- |
| HDL-c |  |  | *0.143 (0.059) p = 0.015* |
| LDL-c |  |  | -0.119 (0.065) p = 0.066 |
| Triglycerides |  |  | *-0.080 (0.058) p = 0.169* |
| Adiponectin |  |  | 0.045 (0.052) p = 0.384 |
| Systolic blood pressure |  |  | **0.076 (0.021) p < 0.0001** |
| Diastolic blood pressure |  |  | **0.070 (0.021) p = 0.001** |
| BMI |  |  | **0.121 (0.044) p = 0.006** |
| BMI (men) |  |  | *0.143 (0.059) p = 0.016* |
| BMI (women) |  |  | 0.102 (0.055) p = 0.063 |
| Height |  |  | **0.127 (0.035) p < 0.0001** |
| Height (men) |  |  | **0.291 (0.072) p < 0.0001** |
| Height (women) |  |  | -0.019 (0.067) p = 0.782 |
| Male pattern balding |  |  | 0.040 (0.105) p = 0.703 |
| Disease outcomes |  |  | Estimate (95% CI) p-value |
| CAD (overall, UKBB) |  |  | 1.06 (0.86 to 1.31) p = 0.59 |
| CAD (men, UKBB) |  |  | 1.14 (0.84 to 1.56) p = 0.41 |
| CAD (women, UKBB) |  |  | 0.99 (0.75 to 1.33) p = 0.97 |
| CAD (overall, C+C4D) |  |  | 0.82 (0.66 to 1.02) p = 0.07 |
| CAD (overall, UKBB+ C+C4D) |  |  | 0.93 (0.80 to 1.09) p = 0.38 |
| Ischaemic stroke (overall, UKBB) |  |  | *1.43 (1.00 to 2.06)*  *p = 0.049* |
| Ischaemic stroke (men, UKBB) |  |  | 1.61 (0.82 to 3.17) p = 0.17 |
| Ischaemic stroke (women, UKBB) |  |  | 0.52 (0.21 to 1.28) p = 0.15 |

Mendelian randomization estimates for high-density lipoprotein (HDL) cholesterol, low-density lipoprotein (LDL) cholesterol, triglycerides (all SD units), adiponectin (µg/ml, log-transformed), systolic and diastolic blood pressure (mmHg), body mass index (BMI, SD units) and height (SD units), male pattern baldness (log odds ratio, positive values favour more balding), coronary artery disease (CAD) and ischaemic stroke in UK Biobank (UKBB) and publicly available data, i.e., CARDIoGRAMplusC4D1000 Genomes-based GWAS (C+C4D). Estimates (standard errors) are changes in the outcome (odds ratios for disease outcomes) per unit increase in log-transformed sex hormone-binding globulin. Genetic associations were measured in men and women unless otherwise indicated. Italics indicates associations at a nominal level of significance without correction for multiple testing (p < 0.05), bold indicates associations after correction for multiple testing (p < 0.05/7 = 0.007).
